# Interfacial Characteristics of HgCdTe Infrared Detectors Grown on Alternative Substrates

**DOI:** 10.3390/s26041132

**Published:** 2026-02-10

**Authors:** Yuanyuan Li, Qingjun Liao, Huihao Li, Jindong Wang, Hao Wu, Zhenhua Ye, Xiaoning Hu, Chun Lin

**Affiliations:** 1 National Key Laboratory of Infrared Detection Technologies, Shanghai Institute of Technical Physics, Chinese Academy of Sciences, Shanghai 200083, China; liyuanyuan7201@163.com (Y.L.);; 2 University of Chinese Academy of Sciences, Beijing 100049, China

**Keywords:** HgCdTe, GaAs substrate, interfacial recombination

## Abstract

To overcome the limitations of CdZnTe substrates for large-format, low-cost HgCdTe infrared focal plane arrays (IRFPAs), the epitaxial growth of HgCdTe films on alternative substrates (e.g., GaAs and Si) has become an important research focus. The lattice mismatch of approximately 14% between the GaAs alternative substrate and the HgCdTe material generates a high density of interfacial defects, such as dislocations and twins. These defects induce a high density of interface states within the near-interface bandgap, resulting in interfacial recombination and consequently limiting device performance. This paper proposes an optimization method for the HgCdTe/GaAs interface that involves substrate removal and surface passivation after the fabrication of GaAs-based HgCdTe infrared (IR) detectors. The GaAs substrate was removed without damage through chemical mechanical polishing (CMP) and selective wet chemical etching. A bromine-based solution (Br_2_–HBr) was employed to eliminate the surface damage layer for interfacial optimization, and a composite dielectric film was deposited to achieve simultaneous surface passivation and optical antireflection. Experimental results on n-on-p devices operating at 80 K demonstrate that after interfacial optimization, the average quantum efficiency across the 3.5–6.1 μm wavelength range increased from 58% to 84% and the blackbody responsivity improved from 8.7 × 10^6^ V/W to 1.6 × 10^7^ V/W. Both quantum efficiency and blackbody responsivity reached levels comparable to those of CdZnTe-based detectors. Numerical fitting based on the carrier diffusion model indicated that interfacial optimization reduced the surface potential by approximately two orders of magnitude, effectively suppressing interfacial recombination.

## 1. Introduction

The increasing scale of infrared focal plane array (IRFPA) detectors is creating a growing demand for large-format, high-performance HgCdTe epitaxial films. Common substrates for epitaxial HgCdTe films include CdZnTe, Si, GaAs, and Ge. Although CdZnTe substrates offer a good lattice match with HgCdTe, making them an ideal choice for epitaxial growth, their high fabrication cost and limited substrate size hinder their application in large-array detectors [1,2,3,4]. Molecular beam epitaxy (MBE) is a vital area of research because it enables the growth of large-area HgCdTe films on alternative substrates, such as Si and GaAs [5,6,7].

Research on interface states of HgCdTe IRFPA detectors primarily focuses on the PN junction region of HgCdTe photovoltaic chips. Surface electron accumulation can induce energy band bending, leading to surface leakage current, which increases the dark current of the detector and reduces its signal-to-noise ratio [8,9]. For back-illuminated HgCdTe photovoltaic detectors, the interface states between the substrate and the HgCdTe absorption layer also significantly impact detector performance. In particular, significant lattice mismatch and differences in the thermal expansion coefficient exist between the alternative substrate and the HgCdTe epitaxial layer. This leads to the introduction of a high density of defects, such as dislocations and twins, at the interface between the epitaxial layer and the substrate during the epitaxial process [10,11,12]. These defects introduce a high density of interface states within the near-interface bandgap of HgCdTe, which act as non-radiative recombination centers for charge carriers. This triggers interfacial recombination, reduces the minority carrier lifetime, and degrades the quantum efficiency of the infrared (IR) detectors. A lattice mismatch of approximately 14% exists between the GaAs substrate and the HgCdTe epitaxial layer [13]. Although a CdTe buffer layer is typically employed to achieve lattice transition [14], the propagation of dislocations induced by the lattice mismatch still results in a high dislocation density in the epitaxial material (in the order of 10^6^ cm^−2^) [15], making it difficult to meet material quality requirements for high-performance detectors.

**Figure 1:**
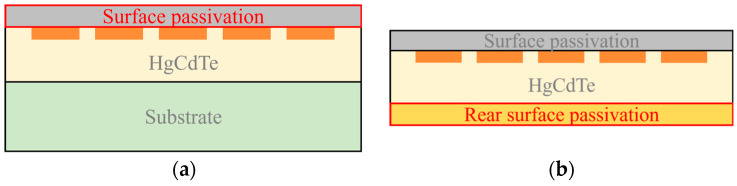
(**a**) Front-side passivation; (**b**) back-side passivation of the epitaxial interface after substrate removal.

The conventional passivation process for HgCdTe photovoltaic devices targets the front side (i.e., the PN junction surface) of the chip, aiming to suppress surface leakage and reduce dark current via deposited or in situ grown passivation films, as depicted in Figure 1a [4,16,17]. For back-illuminated detectors, however, the epitaxial interface between the substrate and the HgCdTe absorption layer—which can be regarded as the “back-side”—also exhibits a high density of defects. This interface remains buried beneath the substrate, posing significant challenges for direct interfacial optimization. To overcome this limitation, this paper proposes an interfacial optimization method for GaAs-based HgCdTe infrared detectors, which involves substrate removal and epitaxial interface passivation to achieve optimization of the HgCdTe/GaAs interface, as illustrated in Figure 1b. The feasibility of this back-side interfacial optimization method for suppressing heteroepitaxial interfacial recombination was systematically investigated. The performance of the detectors before and after interface optimization was comprehensively characterized and analyzed via fitting, confirming the effectiveness of the optimization in significantly enhancing device performance.

## 2. Experiment

A ZnTe/CdTe composite buffer layer was epitaxially grown on a 350 μm thick GaAs substrate with (211)B orientation using a Compact21 MBE system from RIBER, France. The ZnTe layer had a thickness of 10 nm, while the CdTe layer ranged from 5 μm to 10 μm. Subsequently, an 8–10 μm thick HgCdTe film was grown. To validate the performance enhancement achieved via interfacial optimization, the GaAs-based detectors were compared against CdZnTe-based devices, which serve as the industry performance benchmark. This comparison leverages the most mature and standardized process for each substrate: liquid phase epitaxy (LPE) for CdZnTe and MBE for GaAs. The CdZnTe-based HgCdTe IRFPA detector was fabricated using a custom-built horizontal LPE system to grow an 8–12 μm HgCdTe epilayer on a CdZnTe substrate at a growth temperature of 450 °C.

Next, 128-element n-on-p mid-wavelength HgCdTe IR detector chips were fabricated on both of the aforementioned HgCdTe epilayers using the B^+^ ion implantation planar junction process. CdTe/ZnS bilayer passivation was applied to the front side of each chip to suppress surface leakage and control dark current. The chips were then flip-chip-bonded to the readout integrated circuit (ROIC) via indium bumps and mounted onto a sapphire substrate electrode board using epoxy resin adhesive, thereby forming the HgCdTe IRFPA detectors. The detector had a pixel pitch of 240 × 154 μm, a cutoff wavelength of 6.25 μm, and a substrate thickness of 350 μm. It was operated at 80 K.

**Figure 2:**
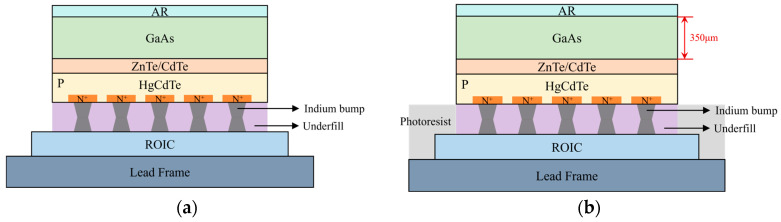

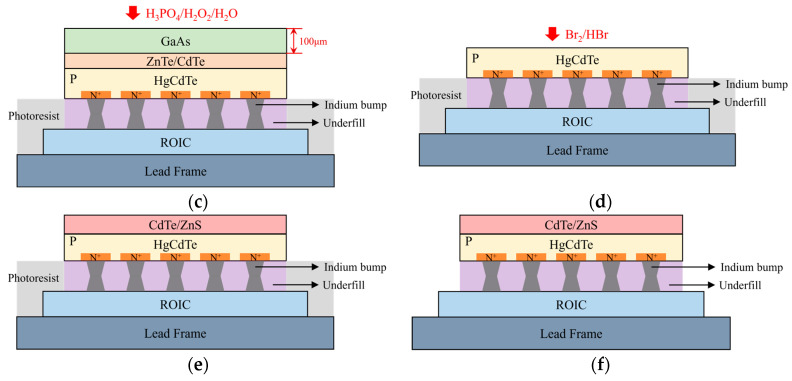
Process flow for substrate removal and interfacial optimization of a GaAs-based HgCdTe detector. (**a**) Initial detector structure with the GaAs substrate; (**b**) Deposition of a protective photoresist layer; (**c**) Substrate thinning via CMP followed by selective wet etching; (**d**) Surface treatment with a Br_2_-HBr solution; (**e**) A CdTe/ZnS composite dielectric film grown on the HgCdTe surface; (**f**) Final device after photoresist removal, ready for characterization.

For the back-side requiring optimization (i.e., the HgCdTe/GaAs epitaxial interface), the GaAs-based detector subsequently underwent the substrate removal and interfacial optimization process illustrated in Figure 2. The performance and spectral response of both the CdZnTe-based and GaAs-based HgCdTe IRFPA detectors (Figure 2a) were characterized using an IRFPA test system from HGH, France. To prevent the failure of the protective mask due to prolonged wet chemical etching, the GaAs substrate of the detector was removed without damage through chemical mechanical polishing (CMP) and selective wet chemical etching. First, a layer of photoresist was coated on the surfaces of both the sapphire substrate electrode board and the ROIC for protection, serving as a mask for the subsequent wet etching process (Figure 2b). The substrate was then uniformly thinned from 350 μm to 100 μm (Figure 2c) through CMP using a PM5 precision polisher from Logitech, UK. The remaining 100 μm GaAs substrate was then selectively wet etched (Figure 2d) using a mixture of phosphoric acid (H_3_PO_4_), hydrogen peroxide (H_2_O_2_), and deionized water (H_2_O) as the etchant. Following this, the HgCdTe surface was treated with a bromine-based solution (Br_2_–HBr) to remove the mechanically damaged and lattice mismatch-induced damaged layer, thereby improving the surface morphology. Subsequently, a CdTe/ZnS composite dielectric film was grown on the HgCdTe surface to provide surface passivation and antireflection functionality (Figure 2e). The photoresist mask was then removed, resulting in a substrate-free HgCdTe detector with a well-defined surface state, as shown in Figure 2f. Finally, the performance of the HgCdTe IRFPA detector with an optimized interface after substrate removal was characterized.

## 3. Results and Discussion

### 3.1. Testing and Analysis of Average Blackbody Responsivity of the Detectors

Performance comparison tests were conducted using an IRFPA test system on GaAs-based HgCdTe IRFPA detectors before and after interfacial optimization processing and on CdZnTe-based HgCdTe IRFPA detectors to evaluate the performance enhancement achieved by the interfacial optimization process. All tested samples were complete focal plane arrays. The detectors were operated under the following conditions: a temperature of 80 K, a bias voltage of approximately −20 mV, an F-number of 4, a blackbody temperature range of 293–308 K, and an integration time of 40 μs.

**Figure 3:**
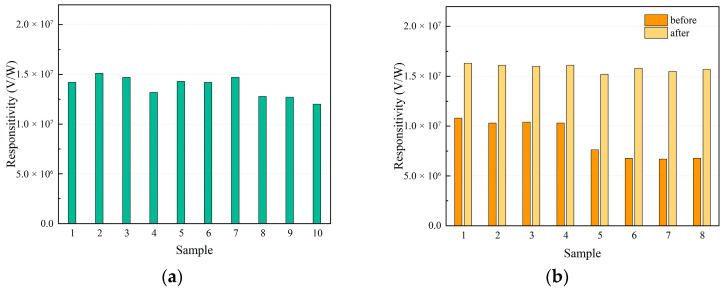
Blackbody responsivity measurement results of (**a**) CdZnTe-based detectors and (**b**) GaAs-based detectors before and after interfacial optimization.

The average blackbody responsivity of ten CdZnTe-based HgCdTe IRFPA detectors was tested, as shown in Figure 3a. The responsivity values of the devices ranged from 1.20 × 10^7^ V/W to 1.51 × 10^7^ V/W, with an average value of 1.4 × 10^7^ V/W across all ten devices. Figure 3b shows a comparison of the responsivity of eight GaAs-based HgCdTe IRFPA detectors before and after interfacial optimization. As shown in Figure 3b, the average blackbody responsivity of these eight original GaAs-based detectors ranged from 6.70 × 10^6^ V/W to 1.08 × 10^7^ V/W. After substrate removal and interfacial optimization, the average blackbody responsivity ranged from 1.52 × 10^7^ V/W to 1.63 × 10^7^ V/W. The alternative substrate interfacial optimization of the HgCdTe film resulted in a significant enhancement of the average blackbody responsivity of the detectors. Comparing the data in Figure 3a and Figure 3b, it is evident that the blackbody responsivity of GaAs-based HgCdTe devices is significantly lower than that of CdZnTe-based HgCdTe devices. After interfacial optimization, the blackbody responsivity of GaAs-based detectors became comparable to, if not superior to, that of the CdZnTe-based detectors.

**Figure 4:**
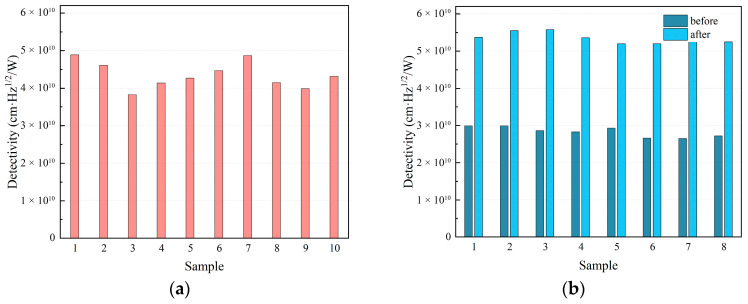
Specific detectivity measurement results of (**a**) CdZnTe-based detectors and (**b**) GaAs-based detectors before and after interfacial optimization.

To quantitatively assess overall device performance, specific detectivity (D*) was introduced as a key indicator of merit. Specific detectivity is defined as the signal-to-noise ratio under the conditions of unit photosensitive area (1 cm^2^) and unit electrical bandwidth (1 Hz). This normalization eliminates the effects of detector size and noise bandwidth, thereby providing a direct and fair comparison of intrinsic detection sensitivity. We measured and calculated the specific detectivity of the aforementioned devices. As shown in Figure 4, the unoptimized GaAs-based detector exhibited an average D* of 2.83 × 10^10^ cm·Hz^1/2^/W. Following substrate removal and interfacial optimization, the average D* increased significantly to 5.39 × 10^10^ cm·Hz^1/2^/W, reaching a value comparable to that of the control CdZnTe-based device (4.35 × 10^10^ cm·Hz^1/2^/W). This result demonstrates that the proposed back-side interfacial optimization effectively enhances the optical response while maintaining excellent noise performance. Consequently, the overall performance of the optimized GaAs-based detector rivals that of its CdZnTe-based counterpart.

### 3.2. Testing and Analysis of Detector Quantum Efficiency

**Figure 5:**
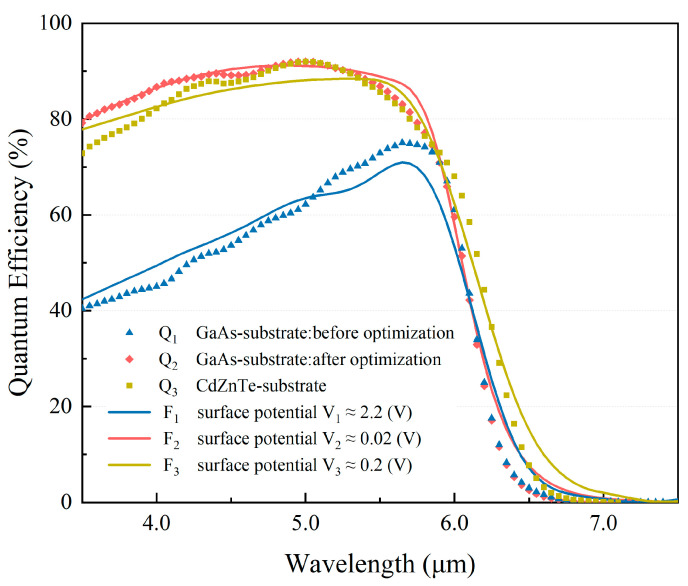
Quantum efficiency comparison and fitting results for GaAs-based detector (before/after optimization) and CdZnTe-based detector.

An improvement in detector responsivity is generally directly linked to an increase in its quantum efficiency. To further investigate how interfacial optimization affects the performance of the GaAs-based detectors, spectral response measurements were conducted on GaAs-based detectors (before and after interfacial optimization) as well as CdZnTe-based HgCdTe detectors. Based on the spectral response data, the quantum efficiency curves of the devices were calculated, as shown in Figure 5. Here, Q_1_, Q_2_, and Q_3_ represent the quantum efficiency curves of the GaAs-based detector before optimization and after optimization and the CdZnTe-based detector, respectively. Following substrate removal, interfacial processing, and passivation and antireflection treatments, the quantum efficiency curve of the GaAs-based HgCdTe detector shifted from Q_1_ to Q_2_. As can be seen from Figure 5, the average quantum efficiency across the 3.5–6.1 μm wavelength range increased from 58% to 84%, which is comparable to the average quantum efficiency of the CdZnTe-based detector (83%) in the same spectral band.

Figure 5 also reveals that changes in the interface states have a more pronounced impact on the quantum efficiency of the GaAs-based detector in the shorter-wavelength region. At λ = 3.7 μm, the quantum efficiency increased from 42% to 83% (Δη=41%); at λ = 5.0 μm, it rose from 62% to 92% (Δη=30%). The enhancement in quantum efficiency is more significant at shorter wavelengths. This is due to the strong dependence of the absorption coefficient of HgCdTe on the wavelength [4]. Short-wavelength photons have a higher absorption coefficient and are primarily absorbed in the near-surface region. The photogenerated carriers produced in this area are more susceptible to interfacial recombination. In contrast, long-wavelength photogenerated carriers are primarily generated within the bulk of the HgCdTe material and are therefore less affected by interfacial recombination.

A comparison of curves Q_1_ and Q_2_ reveals that after interfacial optimization, the peak quantum efficiency wavelength blue-shifted from 5.65 μm to 5.0 μm. This shift is primarily attributed to the thinning of the absorption layer caused by the corrosion of the HgCdTe surface with a bromine-based solution (Br_2_–HBr) during the interfacial optimization process. The reduced thickness lowered the absorption efficiency for long-wavelength photons (>5.0 μm) without a significant performance impact on the short-wavelength band (3.5–5.0 μm). The relative reduction in long-wavelength quantum efficiency resulted in the observed blue shift of the peak wavelength.

### 3.3. Analysis of Surface Potential at the Epitaxial Interface of the Detector

To understand the physical mechanisms underlying the aforementioned experimental results, a numerical model based on carrier diffusion theory was established [18]. The quantum efficiency curves were fitted by solving the Poisson and continuity equations. For p-type HgCdTe material, interface states typically exhibit acceptor-like behavior, capturing electrons and leading to the accumulation of fixed negative charges at the interface. This negative charge induces downward band bending in the near-surface region. Defining the electrostatic potential in the bulk as the reference zero, this band bending corresponds to a negative surface potential (*V*(*d*) < 0). This surface potential creates a potential barrier near the HgCdTe/GaAs interface, which impedes the diffusion of minority carriers toward the PN junction under back-side illumination and significantly reduces the minority carrier lifetime. The total quantum efficiency of the device is collectively influenced by the transmittance of incident light reaching the surface of the absorption layer, the interfacial recombination loss, and the internal quantum efficiency (governed by the carrier transport process). It can be expressed by the following formula [18]:

(1) $$\eta =\frac{\int_{0}^{d_{2}}\left[\exp\left(\frac{qV\left(d\right)-qV}{k_{0}T}\right)\cdot \alpha e^{-\alpha d}\right]dd+\int_{d_{2}}^{d_{1}}\alpha e^{-\alpha d}dd}{\int_{0}^{d_{1}}\alpha e^{-\alpha d}dd}\cdot t\cdot \eta_{0},$$

where *t* is the total transmittance, *d*_1_ is the thickness of the HgCdTe absorption layer, *V* denotes the bulk reference potential (set to zero, *V* = 0), *d*_2_ is the thickness of the interfacial potential barrier region (*d*_2_ < *d*_1_), and *V*(*d*) represents the surface potential, which reflects the degree of band bending. The relationship between the barrier thickness and the surface potential is given by $V(d)=-\frac{qN}{2\varepsilon }{\left(d_{2}\right)}^{2}$, and $\eta_{0}$ is the internal quantum efficiency.

The surface potential *V* is a key parameter characterizing the intensity of interfacial recombination. Its value directly affects the degree of band bending and the minority carrier lifetime at the interface, thereby influencing the quantum efficiency; a greater absolute value of *V* corresponds to stronger interfacial recombination and consequently leads to a reduction in quantum efficiency. The theoretical quantum efficiency curve was fitted to the experimental data over the 3.5–6.2 μm spectral range by adjusting the value of *V* using a least-squares method. The three fitting curves F_1_, F_2_, and F_3_ shown in Figure 5 correspond to the experimental quantum efficiency curves Q_1_, Q_2_, and Q_3_, respectively. The extracted surface potential values are *V*_1_ ≈ −2.2 V (for the GaAs-based detector before interfacial optimization), *V*_2_ ≈ −0.02 V (after optimization), and *V*_3_ ≈ −0.2 V (for the CdZnTe-based detector). The absolute value of *V*_1_ is significantly larger than those of *V*_2_ and *V*_3_, indicating stronger band bending and a higher density of interface states at the unoptimized interface. The fitting results indicate that the absolute value of the surface potential in the GaAs-based HgCdTe device decreased after epitaxial interfacial optimization. This reduction can be attributed to the suppression of defects such as dislocations and twins at the interface, which lowers interface state density, thereby reducing the interfacial recombination rate and enhancing the effective minority carrier lifetime.

As also seen in Figure 5, the absolute value of the surface potential of the CdZnTe-based HgCdTe device is lower than that of the GaAs-based HgCdTe device yet higher than that of the device after epitaxial interfacial optimization. Compared to the GaAs alternative substrate, the CdZnTe substrate exhibits superior lattice matching with the HgCdTe epilayer, resulting in fewer interface states formed at the epitaxial interface. Consequently, the CdZnTe-based HgCdTe detector exhibits superior performance compared to the standard GaAs-based device. However, CdZnTe single crystals may contain defects, such as micro-precipitates, and the LPE process can cause interface irregularities during remelting [19,20]. These factors lead to diffuse scattering and parasitic absorption of optical signals within the CdZnTe substrate under back-side illumination, thereby degrading its transmittance in the IR band. In contrast, the GaAs-based HgCdTe detector with substrate removal and epitaxial interfacial optimization eliminates substrate absorption and scattering. Meanwhile, epitaxial interfacial passivation suppresses interfacial recombination and enhances the collection efficiency of photogenerated carriers.

The above analysis demonstrates, from a physical mechanism perspective, that substrate removal and back-side interfacial passivation directly act on the HgCdTe/GaAs heterointerface. By reducing the interface state density, this approach effectively suppresses interfacial carrier recombination at this location, thereby providing an effective pathway to address the quantum efficiency bottleneck in back-side illuminated heteroepitaxial detectors.

## 4. Conclusions

The epitaxial growth of HgCdTe films on GaAs alternative substrates introduces a high density of interface states due to the lattice mismatch at the epitaxial interface. These states promote the recombination of photogenerated carriers, thereby degrading the performance of the detectors. This study investigated an interfacial optimization approach for GaAs-based IR detectors involving non-destructive removal of the substrate via CMP and selective wet etching while simultaneously growing a composite dielectric film to provide surface passivation and optical antireflection. This approach effectively reduces interface state density and suppresses interfacial recombination at the HgCdTe/GaAs interface. Experimental results demonstrate that both the quantum efficiency and blackbody responsivity of the device significantly improved after interface optimization. The average quantum efficiency across the 3.5–6.1 μm wavelength range increased from 58% to 84%, while the blackbody responsivity improved from 8.7 × 10^6^ V/W to 1.6 × 10^7^ V/W. These performance metrics are comparable to those of lattice-matched CdZnTe-based detectors. Numerical fitting results demonstrated that the absolute value of the surface potential is reduced after interfacial optimization, effectively suppressing recombination at the epitaxial interface. Removing the mismatched substrate and implementing back-side surface passivation provides an effective strategy for inhibiting interfacial recombination in HgCdTe/GaAs structures. This method provides a reliable technological pathway for the fabrication of large-format, high-performance heteroepitaxial HgCdTe IR detectors.

## Data Availability

The data presented in this study are available upon request from the corresponding author.

## Abbreviations

The following abbreviations are used in this manuscript:

IRFPAs	Infrared focal plane arrays
CMP	Chemical mechanical polishing
MBE	Molecular beam epitaxy
LPE	Liquid phase epitaxy
ROIC	Readout integrated circuit
